# The human Müller cell line MIO-M1 expresses opsins

**Published:** 2011-10-20

**Authors:** Margrit Hollborn, Elke Ulbricht, Katja Rillich, Sladjana Dukic-Stefanovic, Antje Wurm, Lysann Wagner, Andreas Reichenbach, Peter Wiedemann, Gloria Astrid Limb, Andreas Bringmann, Leon Kohen

**Affiliations:** 1 Department of Ophthalmology and Eye Hospital, University of Leipzig, Leipzig, Germany; 2 Paul Flechsig Institute of Brain Research, University of Leipzig, Leipzig, Germany; 3 Department of Ocular Biology and Therapeutics, UCL Institute of Ophthalmology, London, UK; 4 Helios Klinikum Aue, Aue, Germany

## Abstract

**Purpose:**

To determine whether the human Müller cell line Moorfields/Institute of Ophthalmology-Müller 1 (MIO-M1) expresses opsins.

**Methods:**

The gene expression of opsins was determined by reverse-transcription PCR (RT–PCR). The presence of opsin proteins was determined by western blotting and immunocytochemistry. The light sensitivity of the cells was examined with imaging experiments using the calcium-sensitive dye Fluo-4.

**Results:**

MIO-M1 cells express glial (glutamine synthase [*GLUL*], vimentin [*VIM*], glial fibrillary acidic protein [*GFAP*], cellular retinaldehyde-binding protein [*RLBP1*], glial high-affinity glutamate transporter [*SLCA1*], aquaporin-4 [*AQP4*], inwardly rectifying potassium channel Kir4.1 [*Kir4.1*]), neuronal (Thy-1 cell surface antigen [*THY1*], heavy neurofilament polypeptide [*NEFH*], microtubule-associated protein 2 [*MAP2*], neurogenic differentiation 1 [*NEUROD1*], neuronal nuclei [*NEUN*]), and neural progenitor markers (Nestin [*NES]*, paired-type homeobox transcription factor [*PAX6*], neurogenic locus notch homolog 1 [*NOTCH1*]). The cells contain mRNA for the following opsins: blue opsin (*OPN1SW*), rhodopsin (*OPN2*), panopsin (*OPN3*), melanopsin (*OPN4*), neuropsin (*OPN5*), and peropsin (*RRH*), as well as for the transducins (guanine nucleotide binding protein [*GNAZ*], alpha transducing activity polypeptide 1 [*GNAT1*], alpha transducing activity polypeptide 2 [*GNAT2*]). The presence of blue opsin and melanopsin was confirmed with immunocytochemistry and western blotting. The immunoreactivity and mRNA of red-green opsin were found in some but not all cultures, while the immunoreactivity for rhodopsin was absent in all cultures investigated. Repetitive stimulation with 480 nm light evoked slow and fast transient calcium responses in the majority of cells investigated, while irradiation with 600 nm light was ineffective.

**Conclusions:**

The human Müller cell line MIO-M1 expresses opsins. This suggests immortalized Müller cells could be used as a cellular source to produce human opsins for their potential application as therapeutic agents in patients with retinitis pigmentosa.

## Introduction

Retinitis pigmentosa includes a group of hereditary disorders characterized by the initial degeneration of photoreceptors [1]. Retinitis pigmentosa is genetically heterogeneous; over 130 genes have been identified that, when mutated, result in severe vision impairment. Different therapeutic strategies were developed in the past to treat retinal dystrophies. Viral replacement of mutated genes has led to the improvement of the phenotypic changes in animal models of retinitis pigmentosa [2]. Other methods represent rather global strategies independent of the underlying mutation. Such strategies include the administration of vitamins, calcium channel blockers, and neuroprotective growth factors [3-5], as well as the transplantation of cells that can adopt photoreceptor characteristics such as pluripotent stem cells and glial cells [6-9].

Postmortem patient eyes with almost complete photoreceptor loss because of retinitis pigmentosa or age-related macular degeneration display preservation of a significant number of inner retinal neurons [1,10-13]. In animal models of photoreceptor degeneration, the remaining inner retina that connects to the brain displays remodeling and degeneration over time [14-18], but can still be functional [19,20]. Thus, electrical stimulation of the remaining inner retina by means of visual prostheses [21,22], or the direct conversion to photosensitive cells of the surviving inner retinal neurons, are further strategies to enhance visual function in retinal dystrophies. It has been shown that the ectopic expression of melanopsin or microbial-type rhodopsin in retinal ganglion cells or bipolar cells of rodless/coneless mice improves the pupillary reflex and the outcome in various irradiance detection tasks [19,23,24].

In addition to the viral transfer of opsin genes, it is conceivable that the transduction of exogenous opsin proteins to inner retinal cells might be helpful in enhancing visual function in retinal dystrophies. The use of human instead of bacterial opsins might decrease the potential risk of inflammatory and immune responses. However, the in vitro and in vivo transduction properties of human opsins have not been demonstrated until very recently. A precondition for the determination of the transduction properties is the production of sufficient amounts of opsins. Approaches to producing human melanopsin by using transformed bacteria or transfected COS-7 cells (a kidney fibroblast cell line of the vervet monkey) in our laboratory or by a commercial provider were not successful (data not shown). Thus, we looked for a human cell line that could be used as a possible source of opsins. We focused our search on the human Müller cell line MIO-M1, which is highly proliferative in culture [25,26]. It has been shown that mammalian Müller cells are capable of expressing neuronal and photoreceptor proteins in animal models of retinopathies, as well as under culture conditions [6-8,27], including proteins of retinal ganglion cells such as Thy-1 [28]. Spontaneously immortalized human Müller cell lines such as MIO-M1 may exhibit progenitor characteristics and may express markers of postmitotic retinal neurons, as well as S-opsin [26]. Here, we show that cultured MIO-M1 cells express various opsins.

## Methods

### Materials

Fluo-4/AM, Pluronic F-127, and Hoechst 33258 were obtained from Molecular Probes (Leiden, Netherlands). All other reagents were obtained from Sigma-Aldrich (Taufkirchen, Germany), unless otherwise indicated. The following antibodies were used: rabbit anti-blue opsin (1:200; Millipore, Schwalbach, Germany), rabbit anti-red-green opsin (1:200; Millipore), monoclonal rat anti-rhodopsin (clone 5G6; 1:200; kindly provided by Stefanie M. Hauck, Dept. of Protein Sciences, Helmholtz Center, Munich, Germany), rabbit anti-melanopsin (1:200; Santa Cruz Biotechnology, Santa Cruz, CA), mouse anti-glutamine synthetase (1:500; clone GS-6; Millipore), mouse anti-glial fibrillary acidic protein (GFAP; 1:200; clone Ga5; Sigma-Aldrich, Taufkirchen, Germany), Cy2-coupled goat antirabbit IgG (1:400; Jackson ImmunoResearch, Newmarket, UK), Cy2-coupled goat antirat IgG (1:400; Jackson ImmunoResearch Laboratories), and Cy3-coupled goat antimouse IgG (1:400; Jackson ImmunoResearch). For western blotting, rabbit antimelanopsin (1:300; Santa Cruz Biotechnology), rabbit anti-OPN1SW (1:700; Santa Cruz Biotechnology), and antirabbit IgG conjugated with alkaline phosphatase (1:3000; Chemicon) were used. The pBSII SK+ OPN4 CDS plasmid was kindly provided by Emma E. Tarttelin (Department of Visual Neuroscience, Division of Neuroscience and Psychological Medicine, Imperial College, London).

### Cell culture

The experiments were performed using the human Müller cell line MIO-M1 [25] and cultured HEK-293 and human retinal pigment epithelial cells. The cells were cultured in tissue culture flasks (Greiner, Nürtingen, Germany) using Dulbecco’s Modified Eagle’s medium (Invitrogen, Paisley, UK) containing 10% fetal bovine serum, glutamax II, and penicillin/streptomycin in 5% CO_2_ at 37 °C. To carry out the calcium imaging experiments and immunostaining, the cells were seeded at 1×10^4^ cells per well in 12-well flat-bottom microtiter plates (Greiner), and allowed to attach for 48 h using medium supplemented with 10% serum. PCR was also performed using RNA extracted from human postmortem retinas. The use of human material was approved by the Ethics Committee of the University of Leipzig, and was performed according to the declaration of Helsinki.

### Total RNA preparation

Total RNA was isolated from MIO-M1 cells with the RNeasy Mini Kit (Qiagen, Hilden, Germany) using 600 µl of RLT buffer for cell lysis, and from neural retinal tissues of human donors (postmortem time up to 24 h) using TRIZOL (Gibco BRL, Paisley, UK). After the addition of the retinal tissues to TRIZOL (1 ml), the tissues were homogenized using Ultraturrax (IKA, Staufen, Germany). Retinal RNA was additionally purified using the RNeasy Mini Kit (Qiagen). The quality of the RNA was analyzed by agarose gel electrophoresis. The A_260_/A_280_ ratio of optical density was measured using the NanoDrop 1000 device (Peqlab, Erlangen, Germany), and was between 2.0 and 2.2 for all RNA samples, indicating sufficient quality.

### Reverse transcription-PCR

After treatment with DNase I (Roche, Mannheim, Germany), cDNA was synthesized from 1 µg of total RNA using the RevertAid H Minus First Strand cDNA Synthesis kit (Fermentas, St. Leon-Roth, Germany). After dilution of the cDNA (1:2) with RNase-free water, PCR was performed using the Taq PCR Master Mix (Qiagen) and the primer pairs described in Table 1. One µl (25 ng template cDNA) of the first-strand mixture and 0.25 µM of each gene-specific sense and anti-sense primer were used for amplification in a final volume of 20 µl. Amplification was performed for 40 cycles with the PTC-200 Thermal Cycler (MJ Research, Watertown, MA). Each cycle consisted of 30 s at 94 °C, 45 s at 58 °C, and 90 s at 72 °C. Negative controls were done by using double-distilled water and samples of reverse transcription performed with total RNA but omitting the reverse transcriptase. In all these controls, no amplicons were detectable (not shown), confirming the specificity of the PCR results. cDNA samples prepared from the RNA of human retinas were used as positive controls. The PCR products were analyzed by agarose (1.7%) gel electrophoresis.

**Table 1:** Primer pairs used for PCR experiments.

**Gene (Accession mRNA/Gene ID)**	**Primer sequence (5′→3′; numbers indicate the localization with respect to the DNA)**			**Amplicon (bp/Tm)**
	**Forward**		**Reverse**	
*ACTB*NM_001101/ 60	_2198_ATGGCCACGGCTGCTTCCAGC _2218_	_2529_CATGGTGGTGCCGCCAGACAG_2509_		237/ 88.0 °C
*RPLP1*NM_213725/ 6076	_2363_GTCAACATTGGGAGCCTCAT_2382_	_2678_AGACCAAAGCCCATGTCATC_2659_		176/ 88.0 °C
*HPRT*NM_000194/ 3251	_15049_TGCTCGAGATGTGATGAAGG_15068_	_26340_TCCCCTGTTGACTGGTCATT_26321_		192/ 82.0 °C
*GAPDH*NM_002046/ 2597	_2619_GCAGGGGGGAGCCAAAAGGGT_2639_	_3122_TGGGTGGCAGTGATGGCATGG_3102_		219/ 86.0 °C
**Opsin**				
*OPN2#*NM_000539/ 6010	_3921_CACCACACAGAAGGCAGAGA_3940_	_4062_CATGAAGATGGGACCGAAGT_4043_		142/ 86.0 °C
*OPN3*NM_014322/ 23596	_373_CACCTCCTCCTGGTCAACAT_393_	_35928_TGTAGGTAATGGCCCTCCAG_35909_		253/ 88.5 °C
*OPN4*NM_033282/ 94233	_4001_GGACCGCTACCTGGTAATCA_4020_	_4851_GGAGGAAGAACACGAAGCAG_4833_		242 / 89.5 °C
*OPN5*NM_001030051/ 221391	_26222_CTGTGGTGCCAACCCTACTT_26241_	_29689_TCATGAATTTCCAGCACAGC_29670_		190/ 83.5 °C
*RRH*NM_006583/ 10692	_14517_CCCTTATTCCATCGTGTGCT_14536_	_16150_TGTCACAGGCATTGTTTGGT_16131_		199/ 82.5 °C
*OPN1SW*NM_001708/ 611	_1911_TCAGCAGCAGGAGTCAGCTA_1930_	_3145_ATGCAAGCTTGGAACTGCTT_2146_		246/ 86.5 °C
*OPN1LW+MW*NM_020061; NM_000513/	TCGCTATCATCATGCTCTGC	GCACTTTTGGCAAAGTAGGC (see below)		247/ 88 °C
*RGR*NM_001012722/ 5995	_7885_CTTCTGGGTTGGGGTCACTA_7904_	_9380_CACTCTTCCCCAGTTTCTGC _9361_		181
**Phototransduction cascade**				
*GNAZ#*NM_002073/ 2781	_52660_TTCATCAACACCTCACTCATCC_52681_	_52842_GATCTCCTTGGTCTCCTTGTTG_52821_		183
*GNAT1*NM_144499/ 2779	_2591_GCTAGTGGAGGACGACGAAG_2610_	_3172_CTCGTAGGTGTTGGGTCCAT_3044_		193
*GNAT2*NM_005272/ 2780	_300_GGAAGCCAAGACTGTCAAGC_319_	_3043_GCACAGCTTGGTTCAGCATA_3024_		216
*ARR3*NM_004312/ 407	_9055_AGAGACTATGTGCGGCTGGT_9074_	_9400_GGGTTCTCCGTGGTAGTGAA_9380_		159
*SAG*NM_000541/ 6295	_19533_GAAGAGCTCCGTGCGATTAC_20832_	_21849_GGCTCCCCATGGAAATAGAT_21831_		159
**Glial marker**				
*GLUL*NM_001033056/ 2752	_6338_TGGGAGCAGACAGAGCCTAT_6357_	_6780_CAGGAATGGGCTTAGGATCA_6761_		240
*VIM*NM_003380/ 7431	_6526_CCCTCACCTGTGAAGTGGAT_6545_	_7116_TTCCAGCAGCTTCCTGTAGGT_7097_		241
*GFAP*NM_002055/ 2670	_4263_ACATCGAGATCGCCACCTAC_4282_	_7470_ATCTCCACGGTCTTCACCAC_7451_		166
*RLBP1*NM_000326/ 9617	_6562_ACAAGTATGGCCGAGTGGTC_6581_	_9840_TCCTCATTCTCCAGCAGCTT_9821_		121
*Kir4.1#*NM_002241/ 3766	_27885_CAAGGACCTGTGGACAACCT_27904_	_28105_GGGATTCAAGGGAGAAGAGG_28086_		221
*AQP4#*NM_001650/ 361	_3244_GGAATTTCTGGCCATGCTTA_3263_	_3479_AGACTTGGCGATGCTGATCT_3450_		226
*SLC1A*NM_001166695/ 6507	_64817_CATGCACAGAGAAGGCAAAA_64836_	_70641_AGAGTCTCCATGGCCTCAGA_70622_		210
**Neuronal marker**				
*NEUN*NM_001082575/ 146713	_380894_CAACGGCTGGAAGCTAAATC_384751_	_386949_GGCTGAGCGTATCTGTAGGC_386920_		264
*THY1#*NM_006288/ 7070	_3198_CTAGTGGACCAGAGCCTTCG_3217_	_3433_TGGAGTGCACACGTGTAGGT_3414_		236
*MAP2*NM_031845/ 4133	_229258_CCAATGGATTCCCATACAGG_229277_	_254576_CTGCTACAGCCTCAGCAGTG_254557_		180
*NEFH*NM_021076/ 4744	_5561_GAGGAACACCAAGTGGGAGA_5580_	_8721_TTCTGGAAGCGAGAAAGGAA_8702_		160
*NEUROD1#*NM_002500/ 4760	_1888_GTTCTCAGGACGAGGAGCAC_1907_	_2040_CTTGGGCTTTTGATCGTCAT_2021_		164
**Progenitor marker**				
*NES#*NM_006617/ 10763	_4438_GCTCCTCTCTCTCTGCTCCA_4457_	_4666_CACCGGATTCTCCATCCTTA_4647_		229
*PAX6*NM_001604/ 5080	_16055_TGCAGATGCAAAAGTCCAAG_16074_	_16348_TTTCCCAAGCAAAGATGGAC_16329_		200
*NOTCH1*NM_017617/ 4851	_37744_ACTGTGAGGACCTGGTGGAC_37763_	_38456_TTGTAGGTGTTGGGGAGGTC_38437_		196

T_m_ represents melting temperature of the PCR product. # represents primer that does not span an intron. Genomic localization for primers: *OPN1LW* (Gene ID 5956), Forward: 10441–10460, Reverse: 12241- 12222; *OPN1MW* (Gene ID 2652), Forward: 9211–9230 (4 mismatches), Reverse: 11011–10992 (1 mismatch).

### Real-time PCR

The cDNA was diluted (1:2) by the addition of 20 µl RNase-free water. Semiquantitative real-time PCR was performed with the Single-Color Real-Time PCR Detection System (BioRad, Munich, Germany) using the primer pairs described in Table 1. The PCR amplification mixture contained 25 ng template cDNA, a specific primer set (0.25 µM each), and 10 µl of iQ SYBR Green Supermix (BioRad) in a final volume of 20 µl. The following conditions were used: initial denaturation and enzyme activation (one cycle at 95 °C for 3 min) and denaturation, amplification, and quantification (45 cycles at 95 °C for 30 s, 58 °C for 20 s, and 72 °C for 45 s). This was followed by a melt curve analysis (81 cycles) to determine the product specificity, wherein the temperature was gradually increased (0.5 °C/cycle) from 55 °C to 95 °C. The fluorescence signal was measured during every cycle. The amplified samples were analyzed by standard agarose gel electrophoresis. The mRNA expression was normalized to the expression level of hypoxanthine phosphoribosyl-transferase1 (*HPRT1*). Similar results were obtained using acidic ribosomal phosphoprotein P1 (*RPLP1*) or glyceraldehyde-3-phosphate dehydrogenase (*GAPDH*) expression for normalization (data not shown). Statistical significance was evaluated with the Mann–Whitney U test. For the calculation of the relative expression changes, we used the fluorescence signal of lower cycles during the log phase of the product formation. Real-time PCR efficiency (E) was calculated according to the equation E=10^[-1/slope]^. The efficiencies were similar in the investigated range between 0.05 ng and 100 ng cDNA for all target genes (panopsin [*OPN3*], 2.14; melanopsin [*OPN4*], 2.21; rhodopsin [*OPN2*], 2.18; peropsin [*RRH*], 2.20; blue opsin [*OPN1SW*], 2.00; *HPRT1*, 2.09; *RPLP1*, 2.04; *GAPDH*, 2.00), with the exception of neuropsin (*OPN5*; 2.62).

### Western blotting

The cells were seeded in 1×10^5^ cells in cell culture flasks (25 cm^2^) in 3 ml complete medium until ~70%–90% confluency was reached. Thereafter, the medium was removed, and the cells were washed twice with cold phosphate-buffered saline (pH 7.4; Invitrogen). Cytosolic and membrane proteins of MIO-M1 cells were extracted using the ProteoJET Membrane Protein Extraction Kit (Fermentas) according to the manufacturer’s instructions. The extracted proteins were concentrated (~1:100) using the Vivaspin 5000-System (Satorius, Göttingen, Germany). Proteins from retinal tissues were extracted with the mammalian cell lysis kit (MCL1; Sigma-Aldrich). Equal amounts of protein (20 µg) were separated by 10% sodium dodecyl sulfate PAGE (SDS–PAGE). Immunoblots were probed with primary and secondary antibodies, and immunoreactive bands were visualized using 5-bromo-4-chloro-3-indolyl phosphate/nitro blue tetrazolium. No specific signals were detected in the negative controls made by omitting the primary antibody or using purified rabbit IgG (Cell Signaling Technologies, Danvers, MA).

### Immunocytochemistry

For immunolabeling, the cultures were fixed in 4% paraformaldehyde for 10 min. After several washing steps in buffered saline, the cultures were incubated in 5% normal goat serum plus 0.3% Triton X-100 (Sigma-Aldrich) in saline for 1 h at room temperature and, subsequently, in a mixture of primary antibodies overnight at 4 °C. After washing in saline plus 0.3% Triton X-100, secondary antibodies and Hoechst 33258 were applied for 2 h at room temperature.

### Calcium imaging

The experiments were performed at 21–23 °C. After incubation of the cells in culture medium containing Fluo-4/AM (10 µM) for 30 min, the cells were either dark adapted for 45 min or stored under room illumination. The stock solution of the dye was made in dimethyl sulfoxide (DMSO) plus Pluronic F-127 (2%). Thereafter, the microtiter plates with the cells were transferred to a recording chamber filled with extracellular solution containing (in mM) 135 NaCl, 3 KCl, 2 CaCl_2_, 1 MgCl_2_, 1 Na_2_HPO_4_, 10 HEPES, and 11 glucose. During the recordings, the cells were superfused with extracellular solution (1 ml/min). Fluo-4 was excited at 480 nm, and the emission was recorded with a band-pass filter between 505 nm and 550 nm. A conventional fluorescence microscope system (Till Photonics, Munich, Germany; consisting of Polychrome IV, SensiCam, and Till Vision Software; microscope: Axioskop; Zeiss, Jena, Germany) was used. Repetitive light stimuli were applied for 10 min; 480 nm light stimuli with a duration of 500 ms were applied with a frequency of one per second, while 600 nm light stimuli with a duration of 18 s were applied every 20 s. During the administration of the 600 nm light stimuli, Fluo-4 was excited with 480 nm light stimuli (duration, 500 ms) every 20 s.

## Results

### Expression of glial, neuronal, and neural progenitor genes

**Figure 1:**
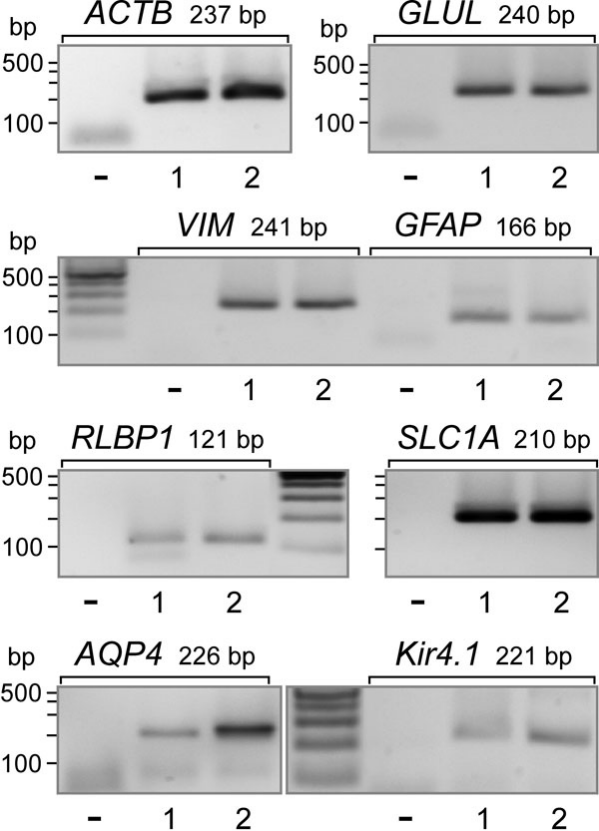
Expression of retinal glial cell marker genes in Moorfields/Institute of Ophthalmology-Müller 1 (MIO-M1) cells. The expression of the following genes was determined with reverse-transcription PCR: glutamine synthetase (*GLUL*), vimentin (*VIM*), glial fibrillary acidic protein (*GFAP*), cellular retinaldehyde-binding protein (*RLBP1*), the glial high-affinity glutamate transporter (*SLC1A*), aquaporin-4 (*AQP4*), and the inwardly rectifying potassium channel Kir4.1 (*Kir4.1*), and β-actin (*ACTB*). The data from two independent experiments (1, 2) are shown. The negative control (-) was done by adding double-distilled water instead of cDNA.

Immortalized human Müller cell lines were shown to express neural progenitor genes and genes for marker proteins of postmitotic retinal neurons [26]. We determined whether MIO-M1 cells contained mRNA for proteins that were characteristic for retinal neuronal, glial, and stem cells. We found that MIO-M1 cells expressed the following marker genes of retinal glial cells (Figure 1): glutamine synthetase (*GLUL*), the glial intermediate filaments vimentin (*VIM*) and glial fibrillary acidic protein (*GFAP*), the cellular retinaldehyde-binding protein (*RLBP1*), the glial high-affinity glutamate transporter (*SLC1A*), aquaporin-4 (*AQP4*), and the inwardly rectifying potassium channel Kir4.1 (*Kir4.1*).

**Figure 2:**
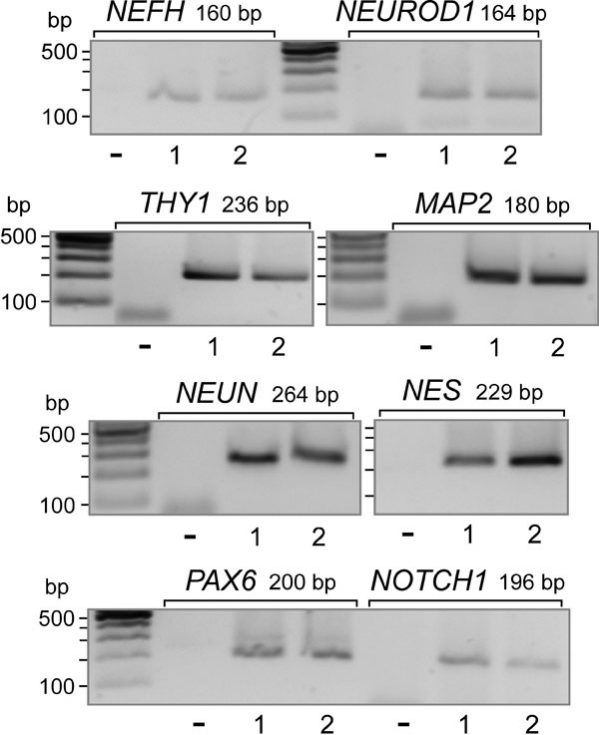
Expression of neuronal and stem cell marker genes in Moorfields/Institute of Ophthalmology-Müller 1 (MIO-M1) cells. The expression of the following neuronal genes was determined with reverse transcription PCR: Thy-1 (*THY1*), neurofilament heavy polypeptide (*NEFH*), neurogenic differentiation 1 (*NEUROD1*), microtubule-associated protein 2 (*MAP2*), and neuronal nuclei (*NEUN*). The expression of the following neural progenitor genes was determined: nestin (*NES*), paired-type homeobox transcription factor (*PAX6*), and neurogenic locus notch homolog protein 1 (*NOTCH1*). The data from two independent experiments (1, 2) are shown. The negative control (-) was done by adding double-distilled water instead of cDNA.

The expression of the following marker genes of mature neurons was observed (Figure 2): the marker of retinal ganglion cells, Thy-1 (*THY1*), the heavy neurofilament protein (*NEFH*), microtubule-associated protein 2 (*MAP2*), the member of the NeuroD family of basic helix–loop–helix transcription factors, neurogenic differentiation 1 (*NEUROD1*), and hexaribonucleotide binding protein 3 or neuronal nuclei (*NEUN*), which is a splicing regulator localized to the nuclei of postmitotic neurons [29]. We found the expression of the following marker genes of neural progenitors in MIO-M1 cells (Figure 2): the intermediate filament nestin (*NES*), the regulator of gene transcription paired box gene 6 (*PAX6*), and Notch homolog 1 (*NOTCH1*). Retinal tissues from postmortem donors contained transcripts for all the glial, neuronal, and progenitor genes examined (data not shown).

### Expression of opsins

**Figure 3:**
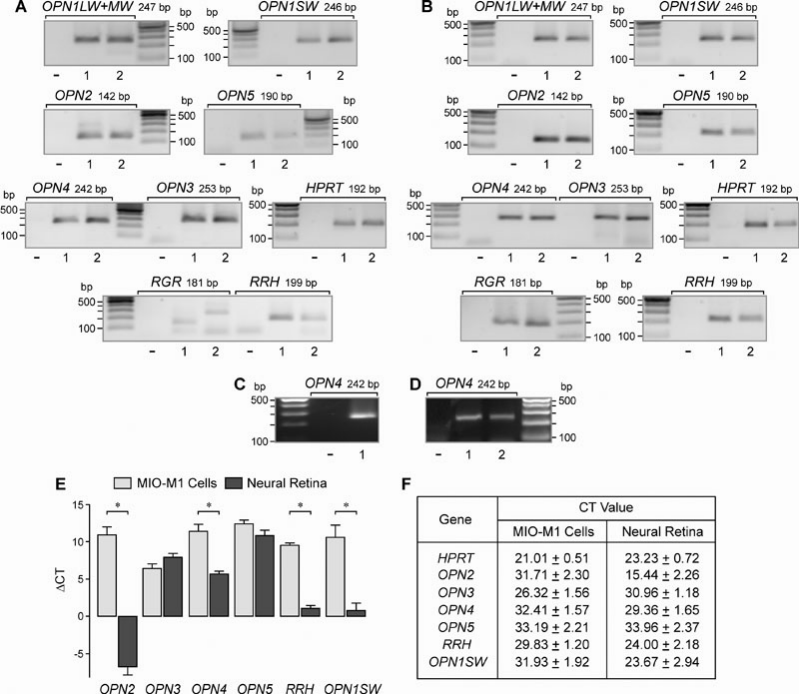
mRNA expression of opsins in Moorfields/Institute of Ophthalmology-Müller 1 (MIO-M1) cells and human retinas. **A**: The expression of the following genes was determined with real-time PCR in MIO-M1 cells and (**B**) in neural retinal tissues from post-mortem donors. Long- and midwave-sensitive cone opsin (*OPN1LW+MW*), shortwave-sensitive cone opsin (*OPN1SW*), rhodopsin (*OPN2*), panopsin (*OPN3*), melanopsin (*OPN4*), neuropsin (*OPN5*), retinal G protein-coupled receptor (*RGR*), and peropsin (*RRH*). In addition, the detection of hypoxanthine phosphoribosyl-transferase (*HPRT*) transcripts is shown. The data from two independent experiments (1, 2) are shown. The negative control (-) was done by adding double-distilled water instead of cDNA. **C**: A plasmid (pBSII SK+*OPN4*) containing the open reading frame of melanopsin (*OPN4*; 1) was used as positive control for the PCR experiments. **D**: MIO-M1 cells that were cultured for 18 h in the absence (1) and presence (2), respectively, of fetal bovine serum (10%) contained transcripts for *OPN4*. **E**: Relative expression level of opsins in MIO-M1 cells and postmortem neural retinal tissues. The data were determined by real-time-PCR. The bars represent the cycle numbers required to detect the transcripts (relative to the cycle number for the detection of *HPRT* mRNA). The data are the means±standard error of the mean (SEM) of seven independent experiments. *P value (p)<0.001. **F**: Mean cycle threshold (CT) values (±SEM) for each gene determined by real-time PCR.

The mRNA expression of opsins in MIO-M1 cells was determined with reverse-transcription PCR. As shown in Figure 3A, we found transcripts for the following opsins in the total RNA extracted from MIO-M1 cells: shortwave (blue)-sensitive cone opsin (*OPN1SW*), rhodopsin (*OPN2*), panopsin (*OPN3*), melanopsin (*OPN4*), neuropsin (*OPN5*), and peropsin (*RRH*). Transcripts for the long- and midwave (red-green)-sensitive cone opsin (*OPN1LW+MW*) were found in three out of seven independent experiments. Transcripts for the retinal G protein-coupled receptor (*RGR*) were apparently not present in the cells (Figure 3A). As the positive control, the presence of transcripts for all opsins examined was confirmed in the total RNA extracted from human postmortem retinas (Figure 3B). Transcripts for melanopsin (*OPN4*) were also found in the RNA transcribed from a plasmid that contained the *OPN4* gene (Figure 3C). Melanopsin (*OPN4*) was expressed in MIO-M1 cells that were cultured in the presence or absence (for 18 h) of serum (10%; Figure 3D). Using real-time PCR, we compared the relative expression levels of various opsins in MIO-M1 cells and retinal tissues. The expression levels of all opsin genes investigated (with the exception of *OPN3* and *OPN5*) were significantly lower in MIO-M1 cells compared to retinal tissues (Figure 3E). In particular, the expression of rhodopsin (*OPN2*) was very high in retinal tissues when compared to MIO-M1 cells. The CT values for each gene determined by real-time PCR are shown in Figure 3F.

### Expression of transducins and arrestins

**Figure 4:**
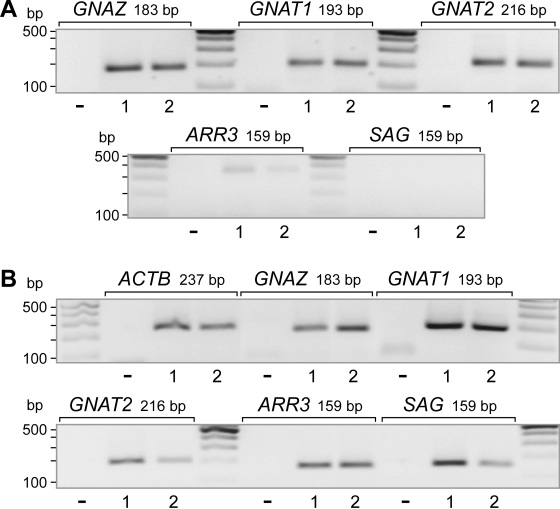
mRNA expression of phototransduction proteins. **A**: The expression was determined with reverse-transcription PCR in Moorfields/Institute of Ophthalmology-Müller 1 (MIO-M1) cells. **B**: In human post-mortem neural retinas. The expression of the following genes was determined with RT–PCR: transducin-α (*GNAZ*), rod-type transducin-α (*GNAT1*), cone-specific transducin-α (*GNAT2*), retinal arrestin (cone arrestin; *ARR3*), and S antigen (rod photoreceptor arrestin; *SAG*). The data from two independent experiments (1, 2) are shown. The negative control (-) was done by adding double-distilled water instead of cDNA.

In photoreceptors, transducins are key components of the phototransduction cascade, acting between opsins and cGMP-phosphodiesterase, while arrestins are involved in the desensitization of the photoactivated transduction cascade. We found that MIO-M1 cells contained transcripts for rod- and cone-type transducins, whereas transcripts for arrestins were not detected (Figure 4A). The total RNA extracted from neural retinal tissues of postmortem donors contained transcripts for transducins and arrestins (Figure 4B).

### Detection of opsin proteins

**Figure 5:**
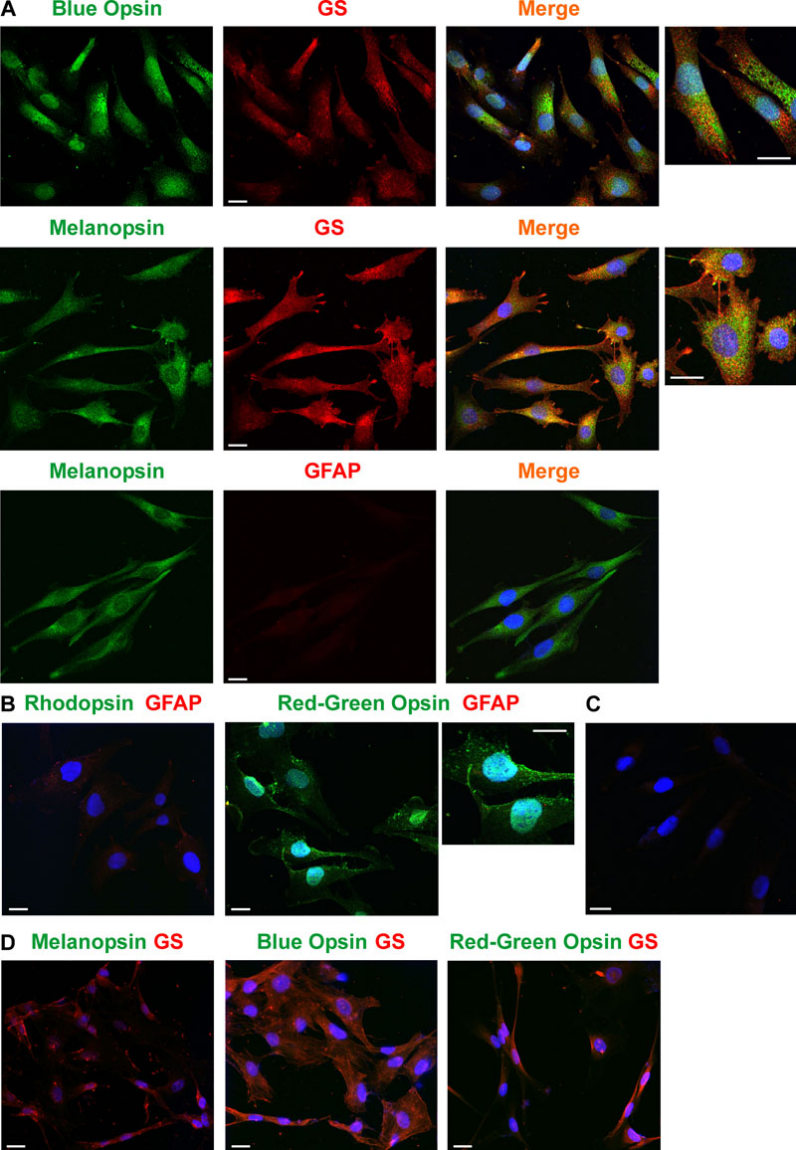
Immunolabeling of opsins in Moorfields/Institute of Ophthalmology-Müller 1 (MIO-M1) cells. **A**: Labeling of blue opsin and melanopsin, respectively, in MIO-M1 cells. The cells were counterstained against glutamine synthetase (GS) and GFAP, respectively. Double labeling yielded a yellow-orange merge signal. **B**: Labeling of rhodopsin and red-green opsin, respectively, in MIO-M1 cells. The cells were counterstained against GFAP. **C**: Control culture of MIO-M1 cells that was stained with secondary antibodies (goat antirabbit IgG and goat antimouse IgG) and without primary antibodies. No nonspecific labeling was observed either, following incubation with goat antirat IgG (not shown). **D:** Immunostaining of HEK-293 cells against melanopsin, blue opsin, and red green opsin, respectively. The cells were counterstained against glutamine synthetase (GS). Cell nuclei were labeled with the DNA dye Hoechst 33258 (blue). Bars are 20 µm.

**Figure 6:**
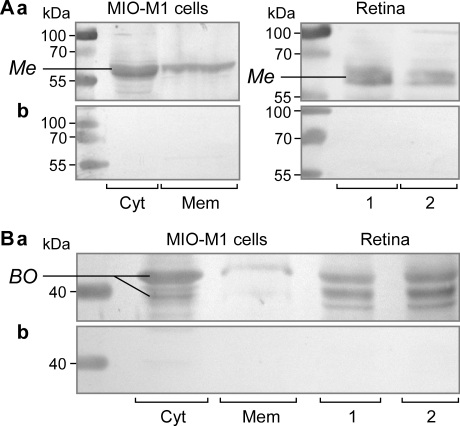
Detection of melanopsin and blue opsin in Moorfields/Institute of Ophthalmology-Müller 1 (MIO-M1) cells and human retinas by western blot analysis. **A**: Melanopsin (*Me*; 65 kDa) was found in the cytosolic (Cyt) and membrane (Mem) fractions of MIO-M1 cells. As the control, the presence of melanopsin was detected in neural retinal tissues from two postmortem donors (1, 2). The blots were stained with (a) and without (b) the first antibody. **B**: Presence of blue opsin (*BO*; 40 and 42 kDa) in the cytosolic (Cyt) and membrane (Mem) fractions of MIO-M1 cells and in two neural retinas (R1, R2), respectively (a). The isotype control (b) was made with purified rabbit IgG.

By using immuncytochemical staining of cell cultures, we found that MIO-M1 cells contained blue opsin and melanopsin (Figure 5). Rhodopsin immunoreactivity was not detected in the cells, while immunoreactivity for red-green opsin was found in some but not all cultures (Figure 5). The presence of melanopsin and blue opsin proteins was confirmed by western blotting. Both proteins were found in the cytosolic and membrane fractions of MIO-M1 cells (Figure 6A,B). As the control, the presence of both proteins was confirmed in protein extracts of neural retinal tissues from postmortem donors (Figures 6A,B). In the case of blue opsin, two major bands (at ~40 kDa and ~42 kDa) were found in the protein extracts of retinal tissues, while in the samples of MIO-M1 cells, the 42 kDa band prevailed (Figure 6B). The presence of several bands could be attributed to posttranslational modifications such as *N*-linked glycosylation, as recently described [30].

As the control, we stained cultured HEK-293 and human retinal pigment epithelial cells with antibodies against red-green opsin, blue opsin, and melanopsin. There was no specific immunolabeling of HEK-293 (Figure 5D) or retinal pigment epithelial cells (not shown) against these opsins.

Though all cells investigated contained transcripts for *GFAP* (Figure 1), some cultures did not display immunoreactivity of GFAP (Figure 5A), whereas other cultures showed very low levels of GFAP immunoreactivity (Figure 5B). However, the cells could be labeled with an antibody against glutamine synthetase (Figure 5A). This is in accordance with a previous study that showed the presence of glutamine synthetase and the absence of GFAP protein in MIO-M1 cells [25].

### Light-evoked calcium responses

**Figure 7:**
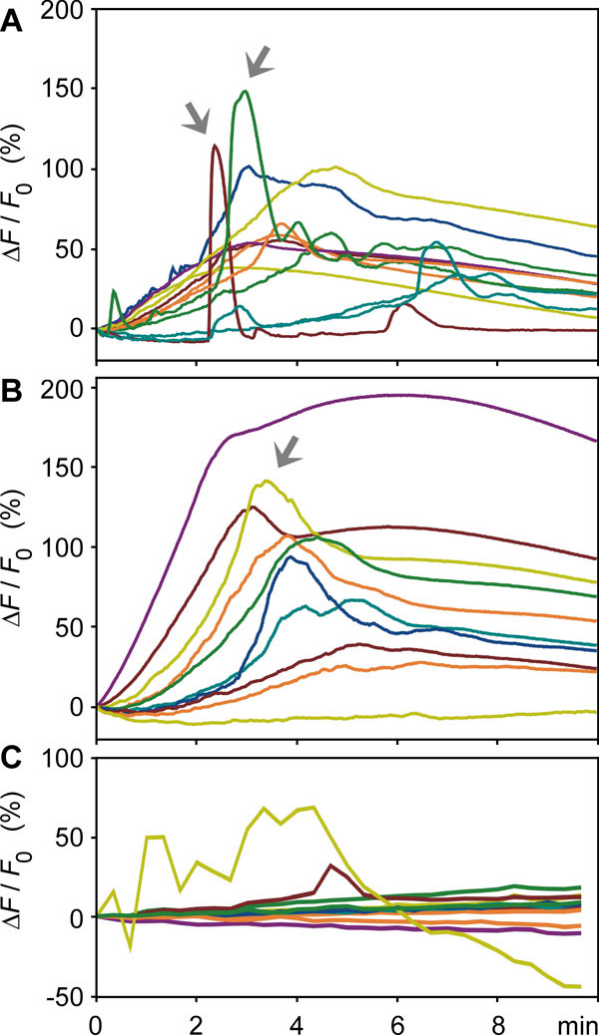
Light-evoked calcium responses in cultured Moorfields/Institute of Ophthalmology-Müller 1 (MIO-M1) cells. **A**: Responses of cells with dark adaptation are shown. **B**, **C**: Responses of cells without previous dark adaptation are shown. The cells were repetitively illuminated with light of 480 nm (**A, B**) and 600 nm (**C**), respectively. The traces represent the light emission alterations of the calcium-sensitive dye, Fluo-4, recorded between 505 nm and 550 nm; an increase in the fluorescence ratio reflects an elevation in cytosolic free calcium. The relative fluorescence change over time was calculated relative to the fluorescence measured at the beginning of the light stimulation (ΔF/F_0_) and is given in percentages. Each trace represents the calcium response of one cell. Arrows indicate fast transient responses.

We found that cultured MIO-M1 cells contained melanopsin and blue opsin (Figure 5 and Figure 6). In a first attempt to prove whether cultured MIO-M1 cells are light sensitive, we performed imaging experiments using the calcium-sensitive dye Fluo-4. We used repetitive stimuli of light with wavelengths of 480 nm and 600 nm, respective experiments. It has been shown in expression studies that human melanopsin has an absorption peak between 420 nm and 430 nm, while murine melanopsin exhibits maximal absorbance around 480 nm [31,32]; almost no light absorbance occurs at 600 nm [33]. Thus, light of a wavelength of 480 nm should predominantly activate melanopsin and rhodopsin, whereas light of a wavelength of 600 nm will predominantly activate red opsin. As shown in Figure 7A,B, repetitive stimulation of the cells with light of a wavelength of 480 nm evoked cytosolic calcium responses in nearly all cells investigated. Light-evoked calcium responses were observed in cells that were dark-adapted for 45 min before the beginning of the recordings (Figure 7A), and in cells that were not dark-adapted before light stimulation (Figure 7B). With the onset of light stimulation, the majority of the responding cells displayed a slowly developing rise in calcium, which reached the maximum after 3–4 min. In most cells, the cytosolic calcium level remained elevated during the recording period of 10 min. In addition to the slow rise in calcium, many of the responding cells also displayed fast transient calcium rises, which occurred with a latency of 2–3 min after the onset of light stimulation (Figure 7A,B). Irradiation of MIO-M1 cells with 600 nm light did not evoke calcium responses in the majority of cells investigated (19 out of 21; Figure 7C). In contrast, irradiation with 480 nm light evoked calcium responses in most of the cells investigated (46 out of 47 cells).

## Discussion

Spontaneously immortalized human Müller cell lines such as MIO-M1 were shown to express neural progenitor genes such as *PAX6* and *NOTCH1*, as well as various genes characteristically for postmitotic retinal neurons [26]. In the presence of extracellular matrix, growth factors, or retinoic acid, these cells can acquire neural morphology [26]. Subretinal or vitreal transplantation of these cells results in translocation of the cells into the retinal parenchyma and the expression of neuronal markers [26]. The present results confirm the previous finding [26] that MIO-M1 cells express marker genes of neural progenitor, glial, and postmitotic neuronal cells (Figure 1 and Figure 2). In addition, we have shown that MIO-M1 cells express various opsins (Figure 3A), contain blue opsin and melanopsin proteins (Figure 5 and Figure 6), and display cytosolic calcium rises in response to repetitive light stimulation (Figure 7). Cytosolic calcium rises were induced in response to 480 nm light (Figure 7A,B) but not to 600 nm light (Figure 7C). These calcium responses might be induced by activation of blue opsin and melanopsin. The absence of calcium responses to 600 nm light irradiation is in agreement with the fact that we did not find transcripts for the red-green-sensitive cone opsin in the majority of RT–PCR experiments done. However, whether the light-evoked calcium responses were mediated by activation of the phototransduction cascade remains to be established in future investigations. The expression of transducins (Figure 4A) does not exclude this possibility.

The kinetics of the slow and fast cytosolic calcium responses in MIO-M1 cells is similar to the kinetics of light-evoked calcium responses in Müller cells, which were recorded in whole-mount and slice preparations of the guinea pig retina [34]. In Müller cells of the guinea pig, the slow light-induced calcium responses are mediated by cellular hyperpolarization, which causes a calcium influx from the extracellular space, whereas the fast light-induced calcium responses are mediated by the release of calcium from intracellular stores, in part after activation of purinergic receptors [34]. Autocrine activation of purinergic receptors after the release of ATP from retinal glial cells has been described as underlying the glial calcium waves in retinal tissue preparations [35] that have a kinetics similar to the fast calcium responses observed in MIO-M1 cells. It could be, but remains to be proven, that the light-induced ion flux that underlies the slow calcium response causes a swelling of MIO-M1 cells, which induces a release of ATP from the cells [36], resulting in the autocrine activation of purinergic receptors and induction of the fast transient calcium responses. Further investigations are required to determine the mechanisms of the light-induced calcium responses in MIO-M1 cells.

In addition to rhodopsin, blue opsin, and melanopsin, we found the expression of panopsin, neuropsin, and peropsin in the neural retina (Figure 3B) and MIO-M1 cells (Figure 3A). The functional roles of these opsins in MIO-M1 cells are unclear. However, because Müller cells were usually found to express various neuronal and photoreceptor proteins under culture conditions [7,27,28], it cannot be ruled out that the expression of opsins is a component of the reactivation of neural progenitor characteristics in gliotic Müller cells, which is probably a precondition of neuronal regeneration by transdifferentiation from Müller cells [6-8,37]. Very little is known regarding the functional roles of panopsin, neuropsin, and peropsin in the retina [38]. Peropsin was described as being expressed in the retinal pigment epithelium [39], and it was suggested that it functions as a retinal isomerase [40]. A similar function of Müller cells, which are implicated in the regeneration of cone photopigments [41], remains to be determined. Panopsin was described as being expressed in different tissues, including the brain, liver, and retina [42]. Neuropsin was found to be localized to chicken amacrine and ganglion cell layer neurons, and it was suggested that it has a functional role in the reception of UV light [43]. It has also been suggested that neuropsin mediates the phototransduction in cells of the paraventricular organ of birds, which regulates seasonal reproduction [44]. However, different opsins are much more widely distributed in the body, and they mediate, for example, the light sensitivity of the skin [45].

Although MIO-M1 cells contain GFAP mRNA (Figure 1), the cells either do not contain GFAP protein or do so at a very low level (Figure 5). The absence of GFAP protein in MIO-M1 cells is in accordance with a previous study [25]. Upregulation of GFAP is a common marker of Müller cell gliosis in situ [46]. However, it has been shown in the chicken retina that Müller cells that increase the expression of GFAP in response to retinal damage do not reenter the cell cycle, while Müller cells that fail to increase the expression of GFAP proliferate and might represent a source of neural regeneration [47]. It is conceivable but remains to be proven that the absence or low expression of GFAP protein in MIO-M1 cells is a precondition of the high proliferative activity of the cells.

It remains to be proven whether transduction of exogenous opsin proteins into the retina in vivo, that is, after intravitreal administration, might represent an approach to improving visual function in retinal dystrophies. It is conceivable that patients may periodically receive intravitreal injections of opsins. Periodical intravitreal injections of agents directed against the vascular endothelial growth factor is a common procedure for treating exudative age-related macular degeneration. To enhance the efficiency of cell transduction, opsins might be coupled to cell-penetrating peptides, that is, through biotinylization. It has been shown, for example, that intravitreal administration of TAT protein-coupled fluorophores or peptides results in preferential uptake by retinal ganglion cells [48,49]. Intravitreal injection of a TAT protein-coupled antiapoptotic protein was shown to inhibit the apoptosis induced by optic nerve lesions in mice [50].

The present results suggest that immortalized human Müller cells could represent a potential source for the production of therapeutic opsins. These cells have a high proliferation rate in culture, and can be frozen and thawed several times without losing their characteristics [26]. However, further research is required to reveal the production conditions, transduction properties, and functional efficiency of human opsins. In addition to a therapeutic purpose, MIO-M1 cells may be used to produce sufficient amounts of opsins for basic research on protein structure and phototransduction [38].

We found that MIO-M1 cells contain at least two opsins, blue opsin and melanopsin (Figure 5 and Figure 6). Various opsins have different advantages and disadvantages in their potential therapeutic use [19]. Melanopsin exerts influence preferentially at high irradiances and responds better to sustained stimuli. Thus, the use of an opsin mixture is conceivable. In addition, microbial-type rhodopsins are preferred under conditions in which the phototransduction cascade is not functional; channel rhodopsin-2 directly forms light-gated ion channels, and its conformational change is caused by reversible photo-isomerization of the chromophore group [51].
